# Bridging two scholarly islands enriches both: COI DNA barcodes for species identification versus human mitochondrial variation for the study of migrations and pathologies

**DOI:** 10.1002/ece3.2394

**Published:** 2016-09-04

**Authors:** David S. Thaler, Mark Y. Stoeckle

**Affiliations:** ^1^ Biozentrum University of Basel CH4056 Basel Switzerland; ^2^ Program for the Human Environment The Rockefeller University New York New York 10065

**Keywords:** DNA barcode, genetic variation, *Homo sapiens*, mitochondrial genome, race, species identification, subspecies

## Abstract

DNA barcodes for species identification and the analysis of human mitochondrial variation have developed as independent fields even though both are based on sequences from animal mitochondria. This study finds questions within each field that can be addressed by reference to the other. DNA barcodes are based on a 648‐bp segment of the mitochondrially encoded cytochrome oxidase I. From most species, this segment is the only sequence available. It is impossible to know whether it fairly represents overall mitochondrial variation. For modern humans, the entire mitochondrial genome is available from thousands of healthy individuals. SNPs in the human mitochondrial genome are evenly distributed across all protein‐encoding regions arguing that COI DNA barcode is representative. Barcode variation among related species is largely based on synonymous codons. Data on human mitochondrial variation support the interpretation that most – possibly all – synonymous substitutions in mitochondria are selectively neutral. DNA barcodes confirm reports of a low variance in modern humans compared to nonhuman primates. In addition, DNA barcodes allow the comparison of modern human variance to many other extant animal species. Birds are a well‐curated group in which DNA barcodes are coupled with census and geographic data. Putting modern human variation in the context of intraspecies variation among birds shows humans to be a single breeding population of average variance.

## Introduction

Science develops as isolated and compact “pebbles,” or islands, of understanding in a vast and unknown sea (Newton 1885). Occasionally, a bridge can be built between islands by identifying areas of correspondence (Singh 1997). Different fields sometimes view the same puzzles from different angles (Adams and Light 2014). In fortunate cases, these different views inform each other and both fields are enriched because hard problems in one island, or field, can be understood by reference to the other. This work identifies such a fortunate case in evolutionary biology.

Two isolated islands of the literature concern themselves with the analysis of mitochondrial DNA sequence: (1) human mitochondrial variation; and (2) DNA barcode analysis. After a brief indication of each subfield, or island of scholarly information, three questions are framed that can be better addressed via their intersection.

The study of human mitochondrial sequence variation informs two subfields: (1) human migration (Ingman et al. 2000; Weissensteiner et al. 2016); and (2) mitochondrial pathologies (Falk et al. 2015; Picard et al. 2015; Murphy et al. 2016). There is an overlap between these two subfields and many workers and resources are involved in both (Ruiz‐Pesini et al. 2004; Lott et al. 2013). In most of the recent human mitochondrial studies, the entire genome is sequenced. So far as we are aware, there is little or no overlap between the analysis of human mitochondrial genetic variation and the use of mitochondrial sequences in the identification and characterization of species and subspecies.

A 648‐base pair (bp) segment of the mitochondrial COI gene has proven effective and has become the dominant standard for the genetic identification of animal species. The sequence of this region is often referred to as a “DNA barcode” (Hebert et al. 2003) (Ratnasingham and Hebert 2013). DNA barcode amplicons are typically obtained by PCR using standardized primer sets; the methods and analysis protocols are robust. There are approximately four and a half million COI barcode sequences in GenBank and/or BOLD (Barcode of Life) databases from multiple individuals in about 250,000 species (*BOLD systems*). Taxonomic domain experts and algorithmic application of the barcode sequence agree for approximately 95% or more of the species in most groups. Controversial or borderline cases that were more closely analyzed turned out to be mostly due to introgression, hybridization, incorrect labeling, or sequence errors (Stoeckle and Thaler 2014). Barcoding works robustly because intraspecies variation is low in most cases. The average pairwise difference among individuals of the same species (APD, equivalent to the term “*π*” which is often used in population genetics) is usually <1%, whereas the distance between even the most closely related animal species is typically 2% or more (Kerr et al. 2007; April et al. 2011; Hausmann et al. 2011). A few animal species have larger APD; most such cases are comprised of distinct genetic clusters corresponding to reproductively isolated populations, which are often recognized as subspecies or races. This study focused on human variation in comparison with that in our closest primate relatives both living and extinct and in birds as exemplars of other animals. Birds were chosen as a group for critical comparison because census and geographic data are most critically curated for a large number of species.

Questions that can be addressed by the intersection of the two fields include:

1. Is the COI DNA barcode region representative of the entire coding mitogenome? The 648‐bp segment was chosen in large part for historical and sociological reasons. Excellent sets of primers were developed and shared. There was and is benefit in using a common region as widely as possible because the new data were more directly commensurable with preexisting datasets of many specimens. Only in the case of modern humans is the entire mitogenome from thousands of individuals available. Comparison of variance in the DNA barcode region to the entire mitochondrial codome of modern humans can address the nagging question of whether or not the COI barcode region is typical and representative.
2. Are synonymous codon substitutions among nearby species functionally and selectively neutral? DNA barcodes among nearby species often differ by 1–2%, almost entirely due to synonymous substitutions. Are different synonymous codons differentially selected in each species? Many thousands of individual human mitochondrial sequences are available whose SNP patterns may be considered in light of health. Among the thousands of human mitochondrial sequences available, approximately 2/3 of the codon positions are found with more than one variant.
3. How does human genetic variation compare to that of the other animals, including but not limited to our closest relatives, among nonhuman primates? Human genetic variation is inherently interesting and is also controversial (Fuentes 2012; Shiao et al. 2012; Templeton 2013; Fujimura et al. 2014; Yudell et al. 2016). It has been suggested that human genetic diversity follows in part from different selection pressures of divergent geographies and social structures (Wade 2015). No other animal species covers so much of the earth (with the possible exception of human commensals). Human societies differ from one another and some of these differences scale to differences in the average behavior of individuals (Gachter and Schulz 2016). Differing genetic selection in different human societies could underlie both effects (Wade 2015). A prediction of this speculation can be tested: Genetic variation is predicted to increase within the species as a whole due to selection for particular alleles in certain environments and/or from reproductive isolation. In either case, the prediction is a relative increase in variance within the modern human species compared to other animal species and a multimodal distribution of that variation in modern humans. DNA barcodes will allow a placing of human variation in the broader context of the entire animal kingdom. This has not been previously possible.

The controversy in comparing human variation to that in other species is consequent to incommensurability (Kuhn 1962). Analysis of modern human variation in isolation or only with respect to closely related species cannot answer an important question: How much modern human variation is there in comparison with the overall animal kingdom and how is the distribution of human genetic variation similar to, or different from, the broad swath of animals? This question has previously been addressed in the context of nonhuman primates (references below) but DNA barcodes allow us to “zoom out” and address the question more broadly in the animal kingdom. Different geographic populations, subspecies, or races imply that the amount of variation in the species would be relatively large or that total variation would have discontinuous features (Templeton 2013). These aspects of human genetic variation can best be understood by comparison with the widest and most complete range of other species including those that do and do not have distinct populations. This comparison is most informative if the same metric can be used across as many species as possible. In this work, we propose and develop the idea that mitochondrial COI DNA barcode analysis provides the best currently available and most broadly applicable metric to quantitatively compare and contrast human genetic variation to that of other animal species. We assert that there is a special value in gaining the ability to “zoom out” and view variation in modern humans in the context of the entire panoply of the extant animal kingdom.

## Materials and Methods

DNA barcodes for modern humans were extracted from human mitogenome sequences that together represent the major mitochondrial haplogroups. Mitogenome datasets in PhyloTree (van Oven and Kayser 2009) were downloaded, totaling 9413 human mitogenomes representing all major African and non‐African lineages (Appendix). An alignment of coding sequences according to GenBank‐defined gene regions was generated in MEGA (Kumar et al. 2004). Barcodes were also bioinformatically extracted from complete mitochondrial sequences of the nearest living and extinct relatives of modern humans: chimpanzees (*Pan troglodytes*), bonobos (*Pan paniscus*), *Homo neanderthalensis,* and *H. sapiens* Denisova. Sequences obtained by focused PCR or bioinformatics extraction from whole genomes are seamlessly compatible. In this way, barcodes and variance of barcode sequences within and across animal species can be aligned and compared.

## Results and Discussion

**Figure 1:**
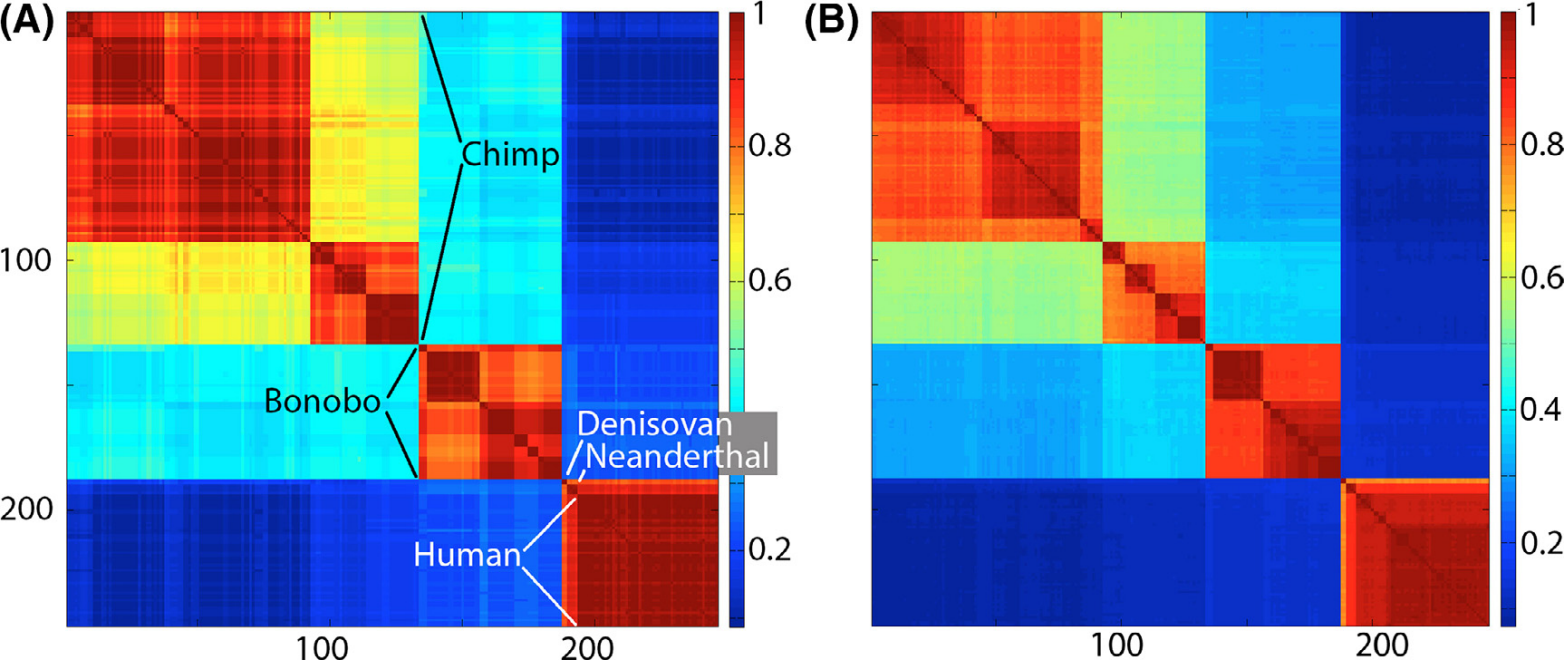
Klee diagram of mitochondrial genetic diversity of humans and our closest living and extinct relatives. The human sequences represent the span of known modern diversity (Ingman et al. 2000). Panel at left is generated from 648‐bp COI barcode sequences and panel at right generated from 5’ half of coding mitogenome (approximately 6 kbp) of the same individuals. The Klee diagram heat map demonstrates greater mitochondrial diversity among chimpanzees and bonobos than among living humans. The COI barcode diagram accurately represents the genetic diversity generated with the coding mitogenome (see also Appendix Fig. A2).

Intra‐ and interspecies variance can be visually compared and contrasted with a Klee diagram: a heat map in which sequences are arrayed against each other so that every sequence is compared with every other one and each intersection of sequences is color‐coded to show the pairs’ similarity (Sirovich et al. 2009). The sequence order is the same on *x*‐ and *y*‐axes and is determined objectively by a tree algorithm, that is, without prior labeling of suspected species or subspecies (Stoeckle and Coffran 2013). A Klee diagram for modern humans and our nearest living neighbor species is shown (Fig. 1). The left panel in which the species are labeled is based completely on the 648‐bp DNA barcode segment. The right‐hand panel is the same set of comparisons using the 5’ half of the entire mitogenome for the same set of organisms. (Limitations of available computing power made it impractical to do the analysis with the entire mitogenomes in one go. Separate runs of the 5’ and the 3’ halves of these mitogenomes are presented side by side in the Appendix.)

The size of the square for each species reflects the number of sequences. In the case of chimpanzees (*n* = 133), bonobos (*n* = 55), *H. sapiens* Denisova (*n* = 2), and *H. neanderthalensis* (*n* = 4), all mitogenomes available at the time of analysis were included. It is impractical in this format to include all 9413 modern human barcodes because the modern human square would dwarf the others. The 53 modern human sequences utilized in this analysis represent the extremes of known modern human diversity, including African, European, Asian, Polynesian, and New World lineages (Ingman et al. 2000). Distinct clusters are evident within the species of chimpanzees and bonobos, which largely correspond to subspecies or regional populations (Becquet et al. 2007; Kawamoto et al. 2013). In contrast, the variation among modern human is more continuous in sequence space (Templeton 2013) and the span of sequence diversity within modern humans is small in comparison with that within our nearest living neighbor species. Denisovans and Neanderthals are distinct from modern humans. However, Figure 1 shows greater differences among populations of modern chimpanzees than among modern humans inclusive of representatives of the extinct forms of *H. sapiens* Denisova and *H. neanderthalensis*. The Klee‐like geometric form of this figure, nonoverlapping squares with sharp edges, arises algorithmically from the sequence data.

No claim of originality is made for the conclusions evident from the comparison of humans to nonhuman primates in Figure 1. References cited above made the key points – the species of modern humans harbors less diversity than that within extant nonhuman primates – prior to the present analysis. The purpose of Figure 1 is to test the DNA barcode method against prior work, as well as against a similar analysis conducted on a much larger sequence (ca. 12 and 25 times larger for half or the entire mitogenome), and confirm that this “bare bones” or, arguably, simple and elegant approach reaches the same conclusions as previously reached by others who used more complex datasets and more statistically intensive methods. Intragenomic studies of the relative power of selection and linkage along each chromosome may be seen as precise articulations of what John Thompson poetically refers to as ripples or foam on the evolutionary sea (Thompson 2013). This paper asserts that DNA barcodes capture deeper evolutionary currents. In addition to simplicity and transparency, the DNA barcode approach to species variance has the advantage of broad applicability. It provides a metric for the comparison of variance in humans with all other animal species for which COI DNA barcodes are available from multiple individuals. An example is given below in which variance in primates is compared with variance in two groups of bird species.

**Figure 2:**
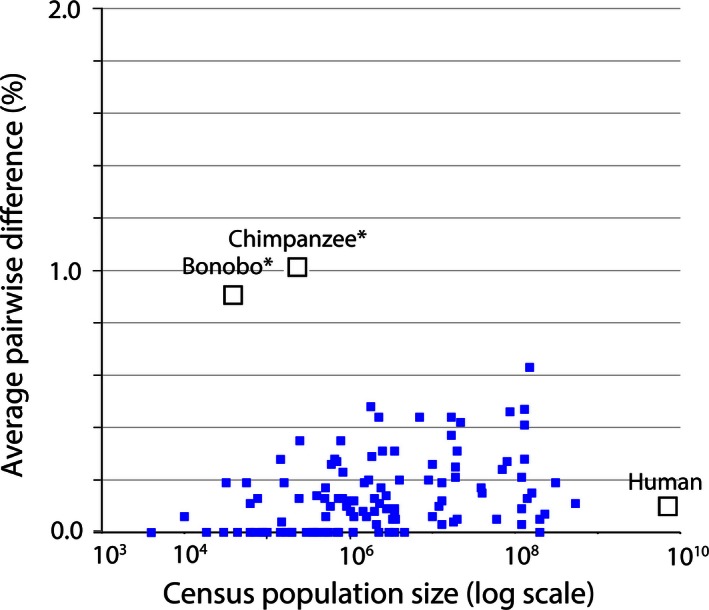
Mitochondrial genetic diversity, represented as average pairwise difference of COI barcodes, in relation to census population size in humans, chimpanzees, and bonobos compared to a well‐characterized set of birds (Stoeckle and Thaler 2014). Mitochondrial genetic diversity in humans is about 0.1%, less than that of many bird species, despite having more than 10‐fold greater population than the most abundant bird in this dataset. Chimpanzees and bonobos have much smaller population sizes than humans, but conspicuously higher diversity, consistent with reproductively isolated subgroups.

Although other effects can complicate (Stewart et al. 1990), neutral variance, calculated as APD, is predicted to increase as a function of population size (Luria and Delbruck 1943; Hedrick 2011). APD yields a single number for each species independent of the number of sequences that are processed. The APD for modern humans was calculated with 9413 barcode sequences. For most species, between 5 and 40 individual barcodes are available. Birds are especially suitable for the analysis of variance as a function of population size because they are the only well‐studied large group for which actual census sizes are known (Fig. 2). The bird analysis in Figure 2 is taken from a previous study that considered apparent cases of introgression, sequencing errors, and misassignment. Curation in this group of avian DNA barcodes has been explained and documented (Stoeckle and Thaler 2014).

The population size of modern humans is approximately ten times that of the most populous bird species in the dataset analyzed, yet the APD of both is 0.1%. Many bird species with smaller population sizes have a larger APD.

Chimpanzees and bonobos are conspicuous in Figure 2 as species with a high APD made all the more striking by their relatively small census population size, but consistent with subspecies or isolated breeding populations (Becquet et al. 2007) (Kawamoto et al. 2013). Nothing about being a primate, apparently, preordains a small mitochondrial APD. Bonobos are reported to have a small amount of nuclear diversity (Prado‐Martinez et al. 2013) in contrast to the high mitochondrial APD. The lack of correlation of genetic variance with population size and the selective forces that keep mitochondrial sequence variation low in animal species remain intriguing evolutionary questions. A low APD for modern humans is consistent with paleontological, anthropological, and historical evidence for a young species that originated within the last 200,000 years and whose population and range expanded dramatically over the last 50,000 years (Henn et al. 2012).

DNA barcodes provide a unique perspective into living diversity because they represent the densest (most individuals per species) and taxonomically broadest sampling of species‐level differences currently available. DNA barcodes are the only sequence information available for multiple individuals in tens of thousands of species. In principle, there are potential pitfalls to using only a short sequence. However, this study showed that human DNA barcode variation is representative of the coding mitogenome as a whole (Fig. 1, and Appendix). Entire mitogenomes increase the resolution of comparisons proportionally to the increased amount of data; however, the DNA barcode region alone – all that is available for most specimens in most species – was here shown representative of the result when whole mitogenomes were compared. The most comparative data are available for modern humans but a similar pattern where whole mitochondrial genomes yield “more of the same” as DNA barcode analysis alone is also shown for other species in the Appendix. It is impossible to say in a logical sense that for cases in which the whole genomes from multiple individuals are not available that the result would always be the same. However, we have found no exceptions and the stereotyped nature of the mitochondrial genome in animals makes the weight of evidence strong that the COI barcode approach developed in this work is indeed a universal or near‐universal fact for the extant animal kingdom. There are times in science when the best is the enemy of the good. A purist bias should not preclude the most widely applicable and informative analysis that can be done at the present time. DNA barcodes can be used to compare variance within and among all animal species, right now. Critical comparison of the barcode region with whole mitogenomes gives high confidence that conclusions from the shorter sequence apply well to the whole. Despite dramatically decreasing costs of sequencing, a “thousand genome project” seems unlikely to be carried out in tens of thousands of other animal species (1000 Genomes Project Consortium et al. 2012). If and when they are, it will be of interest to contrast their results with the COI DNA barcode analyses.

Mitochondrial and nuclear genomes are subject to a Venn diagram of selective forces, that is, some overlap and others do not, such that variance in the two genomes is not always the same. Mitochondrial inheritance is uniparental and the entire mitochondrial genome forms a single “take it or leave it” linkage group (Neher 2013). In contrast, selection in the nucleus differs depending on the gene and locus (Corbett‐Detig et al. 2015). As one example, susceptibility to childhood infectious disease is expected to exert a powerful selection only on a subset of genes (Alcais et al. 2010). Mitochondria are also subject to differential selection in some cases also by infection, for example, Wolbachia infection can lead to mitochondrial divergence in wasps (Xiao et al. 2012). However, selection on the two genomes, mitochondrial and nuclear, also overlaps. The most obvious source of overlap arises from survival and reproduction of the individual organism as a unit of selection (Buss 1987; Gould 2002). The overlapping component of selection would be most consistent with a similar density of neutral SNPs in the mitochondrial and nuclear genomes (Shen et al. 2013).

Despite advances in sequencing and data analysis, nuclear genomes from multiple individuals of multiple species may never approach the millions of mitochondrial COI DNA barcode sequences that are already available and continuing to increase with valuable contributions from citizen science (Geiger et al. 2016). This work shows that any mitochondrial protein‐encoding gene should work as well at least in a “local” sense for species identification and also for the determination of variance in the population. Other things being equal, there is an additional comfort in using the same locus in as many organisms as possible. The COI DNA barcode 648‐bp segment is favored only because there are the most data. There is nothing biologically more optimal about COI compared to any other protein‐encoding segment of the animal mitochondrial genome. Occasionally, a particular group may be more easily analyzed with different primers if, for example, a nuclear pseudogene is amplified with the primers intended for the mitochondrial segment (Lemos et al. 1999). When comparing some groups with others, there may be indels (insertion/deletions) in some genes. In these cases, it may be preferable to use genes that introduce the smallest problems due to indels.

The foremost genetic characteristic of modern humans seen through a comparison of DNA barcode variation with other animal species is that of modest mitochondrial sequence variation made remarkable in light of relatively large population size and wide dispersion. By “zooming out” to examine modern humans as one star in the galaxy of the animal kingdom, our species is seen to have the low variance that is absolutely typical for an animal species with a single breeding population.

## Conflict of Interest

None declared.

## Figures and Tables

**Figure A1:**
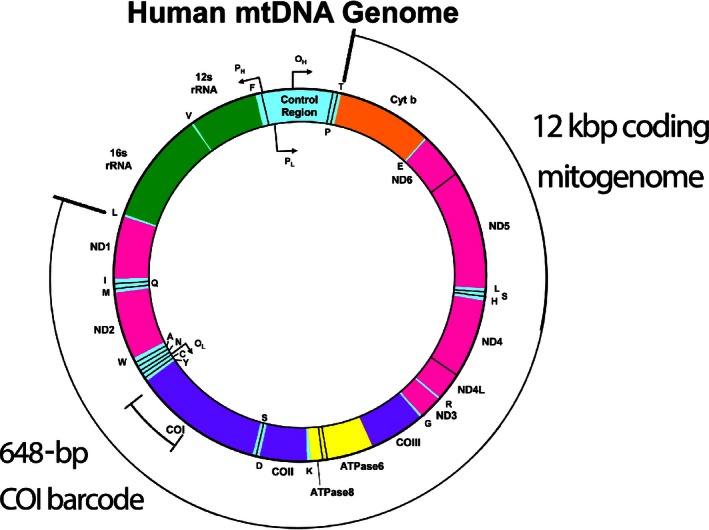
Human mitogenome map with analyzed regions indicated (adapted from www.mitomap.org).

**Figure A2:**
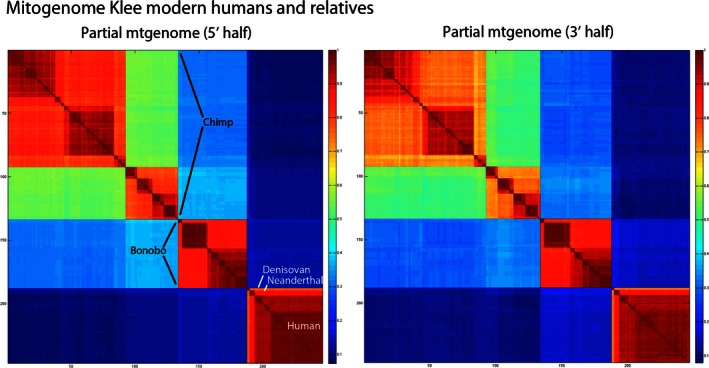
Klee diagram of entire 5’ and 3’ halves of representative human, chimpanzee, and bonobo mitochondrial genomes. The analysis was split due to sequence length computing limitations; the two halves generate essentially identical Klee diagrams. The COI barcode region is contained in the 5’ half.

**Figure A3:**
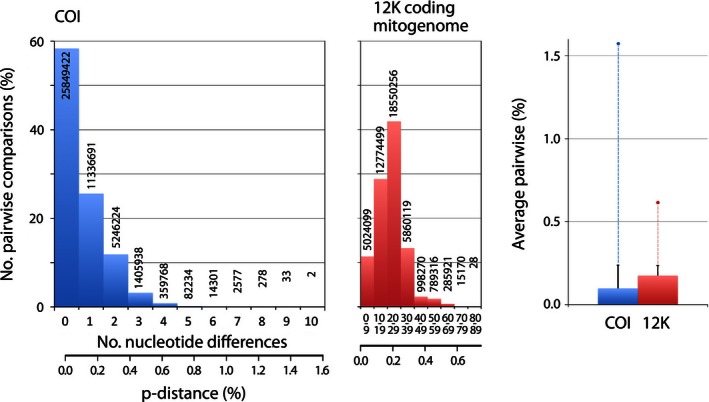
COI barcode (648 bp) variation is representative of coding mitogenome (12 Kbp) variation. COI barcodes and coding mitogenomes were extracted from 9413 complete mitogenomes representing the known range of human diversity (Appendix Table A1). Scale is percent of comparisons; numbers of comparisons (total = 88,604,569) shown on or above each column. Average pairwise differences are at right; whisker and dot indicate standard deviation and maximum value, respectively.

**Figure A4:**
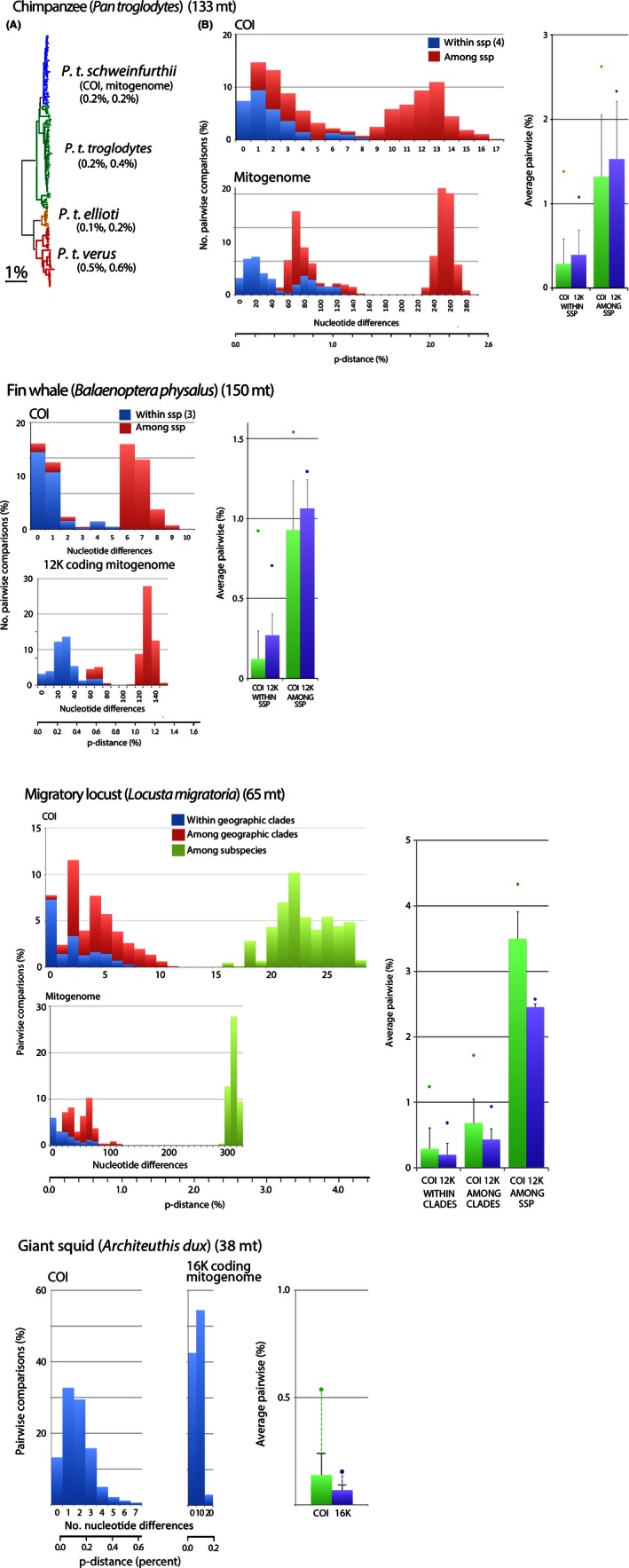
Additional evidence in animal species that COI barcode pairwise differences can be used as metric for 12 Kbp coding mitogenome pairwise differences. Number of complete mitogenomes analyzed is shown in parentheses.

**Figure A5:**
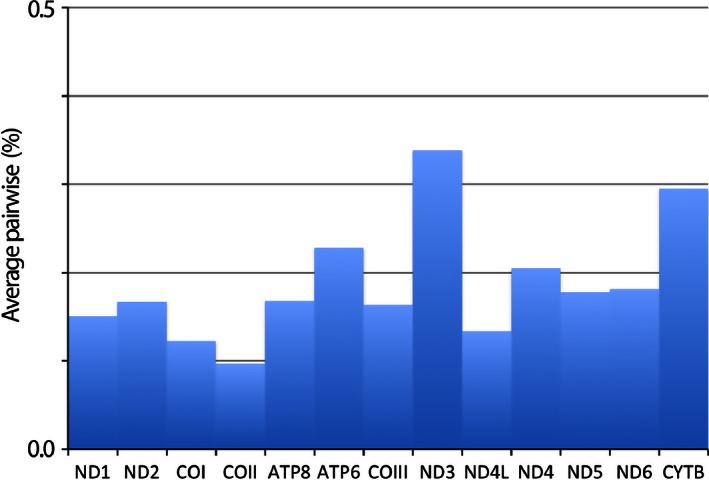
Human nucleotide diversity is similar across entire coding mitogenome. Based on 9413^2^ comparisons, average APD is 0.2%, range 0.1–0.3%.

## Further evidence that the COI DNA barcode supports a universal metric for mitochondrial genetic variance in animal species: COI barcode variance predicts variance of the entire mitochondrial genome in extant humans, other primates, fin whales, birds, locusts, and squid

### Abstract of Appendix

The amount of variation within a biological group, for example, within a species or a population, is an important property of that group. It helps our understanding of the overall structure of the living world to have quantitative measures of variation within and among as many species and populations as we can. For most animal species and populations, only the COI DNA barcode sequence of 648 bp is available from multiple individuals. The question we address here is whether species variation as determined by the COI DNA barcode from multiple individuals is the same as variation determined by the entire mitochondrial genome sequence or by the mitochondrial codome, that is, the portion of the mitochondrial genome that codes for proteins. In the main body of the paper, we show that for a large (>9000 individuals) sample of extant humans, the DNA barcode and the entire mitogenome sequences show a similar variance. The implication that variation in extant humans is spread approximately evenly around the mitochondrial genome is further explored in this Appendix. Furthermore, the comparative analysis of DNA barcodes to the entire mitochondrial genome and/or mitochondrial codome is extended in the Appendix to more species including fin whales and migratory locusts. The inference is strong that variance in the COI DNA barcode alone can be taken as a representative measure of variance in the mitochondrial codome throughout the animal kingdom.

**Table A1:** Human mitogenome datasets. Files were downloaded from PhyloTree.org Build 16 (19 February 2014)

Dataset	No. individuals	Populations
Behar et al. (2012)	4263	Mostly Western Eurasian
Family Tree	1519	Mostly Western Eurasian
Zheng et al. (2012)	910	413 European, 313 African, 184 N American
Duggan et al. (2014)	795	795 Oceanian
Tanaka et al. (2004)	672	672 Japanese
Chandrasekar et al. (2009)	641	641 Indian
Herrnstadt et al. (2002)	560	435 European, 56 African, 52 Native American, 17 Asian
Ingman et al. (2000)	53	African, European, Asian, New World
Total	9413	

**Table A2:** APD in avian species used to generate Figure A2 (dataset from Stoeckle and Thaler 2014)

Species without geographically structured or hybrid clusters				
Latin name	No. of individuals	Ave K2P %	No. of clusters	World pop
*Acanthisflammea*	18	0.15	1	160,000,000
*Actitishypoleucos*	17	0.14	1	2,750,000
*Actitismacularia*	11	0.04	1	150,000
*Agelaiusphoeniceus*	14	0.41	1	130,000,000
*Aphrizavirgata*	2	0.00	1	70,000
*Arenariainterpres*	12	0.17	1	510,000
*Bartramialongicauda*	3	0.13	1	500,000
*Calcariuslapponicus*	13	0.47	1	130,000,000
*Calidris alba*	9	0.28	1	660,000
*Calidrisbairdii*	6	0.00	1	300,000
*Calidriscanutus*	9	0.00	1	1,100,000
*Calidrisferruginea*	4	0.29	1	1,850,000
*Calidrisfuscicollis*	17	0.12	1	1,120,000
*Calidrismaritima*	3	0.00	1	200,000
*Calidrismauri*	17	0.09	1	3,500,000
*Calidrismelanotos*	17	0.11	1	62,500
*Calidrisminuta*	5	0.08	1	1,450,000
*Calidrisminutilla*	16	0.27	1	700,000
*Calidrisptilocnemis*	2	0.00	1	145,000
*Calidrispusilla*	3	0.00	1	2,260,000
*Calidristemminckii*	3	0.00	1	735,000
*Calidristenuirostris*	3	0.00	1	380,000
*Cardellinacanadensis*	9	0.20	1	4,000,000
*Cardellinarubifrons*	2	0.00	1	700,000
*Cardinaliscardinalis*	11	0.21	1	120,000,000
*Columba livia*	29	0.09	1	120,000,000
*Gallinagodelicata*	10	0.08	1	2,000,000
*Gallinagogallinago*	21	0.08	1	3,500,000
*Gallinago media*	4	0.10	1	585,000
*Gallinagomegala*	3	0.00	1	62,500
*Gallinagoparaguaiae*	5	0.08	1	1,025,000
*Gallinagostenura*	6	0.06	1	512,500
*Geothlypisformosus*	8	0.09	1	2,800,000
*Geothlypisphiladelphia*	8	0.44	1	17,000,000
*Geothlypistolmiei*	11	0.10	1	12,000,000
*Geothlypistrichas*	24	0.46	1	87,000,000
*Helmitherosvermivorum*	4	0.23	1	830,000
*Junco hyemalis*	60	0.05	1	200,000,000
*Limicolafalcinellus*	4	0.00	1	87,000
*Limnodromusgriseus*	6	0.13	1	245,000
*Limnodromusscolopaceus*	6	0.00	1	500,000
*Limnothlypisswainsonii*	2	0.00	1	90,000
*Limosafedoa*	3	0.00	1	171,500
*Limosahaemastica*	3	0.13	1	77,000
*Limosalapponica*	7	0.06	1	1,124,000
*Lymnocryptesminimus*	6	0.12	1	1,000,000
*Micropalamahimantopus*	3	0.13	1	820,000
*Mniotiltavaria*	16	0.05	1	20,000,000
*Molothrusbonariensis*	10	0.00	1	200,000,000
*Numeniusamericanus*	2	0.19	1	161,000
*Numeniusarquata*	6	0.10	1	917,500
*Numeniusmadagascariensis*	5	0.19	1	32,000
*Numeniustahitiensis*	6	0.06	1	10,000
*Oporornisagilis*	4	0.20	1	1,700,000
*Oreothlypiscelata*	23	0.27	1	80,000,000
*Oreothlypiscrissalis*	2	0.00	1	30,000
*Oreothlypisluciae*	3	0.00	1	3,000,000
*Oreothlypisperegrina*	16	0.24	1	70,000,000
*Oreothlypisvirginiae*	4	0.00	1	1,100,000
*Parkesiamotacilla*	3	0.00	1	360,000
*Parkesianoveboracensis*	25	0.21	1	19,000,000
*Passer domesticus*	39	0.11	1	540,000,000
*Phalaropusfulicaria*	8	0.00	1	200,000
*Phalaropuslobatus*	33	0.05	1	3,600,000
*Phalaropus tricolor*	2	0.19	1	1,500,000
*Philomachuspugnax*	23	0.11	1	2,300,000
*Protonotariacitrea*	6	0.06	1	1,600,000
*Scolopax minor*	5	0.00	1	3,500,000
*Scolopaxrusticola*	11	0.04	1	18,018,750
*Seiurusaurocapilla*	21	0.42	1	22,000,000
*Setophagaamericana*	13	0.03	1	13,000,000
*Setophagacaerulescens*	12	0.03	1	2,100,000
*Setophagacastanea*	7	0.20	1	9,000,000
*Setophagacerulea*	3	0.26	1	600,000
*Setophagacitrinia*	5	0.00	1	4,600,000
*Setophagacoronata*	32	0.28	1	130,000,000
*Setophaga discolor*	5	0.31	1	3,500,000
*Setophagadominica*	4	0.48	1	1,800,000
*Setophagafusca*	7	0.06	1	10,000,000
*Setophagagraciae*	3	0.13	1	2,000,000
*Setophagakirtlandii*	3	0.00	1	4000
*Setophaga magnolia*	26	0.15	1	40,000,000
*Setophaganigrescens*	7	0.17	1	2,400,000
*Setophagaoccidentalis*	5	0.31	1	2,500,000
*Setophagapalmarum*	13	0.12	1	13,000,000
*Setophagapennsylvanica*	14	0.25	1	19,000,000
*Setophagapinus*	4	0.19	1	13,000,000
*Setophagapitayumi*	8	0.31	1	20,000,000
*Setophagaruticilla*	22	0.17	1	39,000,000
*Setophagastriata*	22	0.05	1	60,000,000
*Setophagatigrina*	8	0.44	1	7,000,000
*Setophagatownsendi*	10	0.37	1	17,000,000
*Setophagavirens*	11	0.26	1	10,000,000
*Spizellapasserina*	21	0.07	1	230,000,000
*Sturnus vulgaris*	23	0.63	1	150,000,000
*Tringabrevipes*	4	0.00	1	44,000
*Tringaerythropus*	8	0.28	1	145,000
*Tringaflavipes*	12	0.14	1	400,000
*Tringaglareola*	17	0.05	1	3,300,000
*Tringaincana*	3	0.00	1	18,500
*Tringamelanoleuca*	9	0.00	1	100,000
*Tringanebularia*	11	0.35	1	780,000
*Tringaochropus*	9	0.44	1	2,250,000
*Tringasemipalmatus*	8	0.35	1	250,000
*Tringastagnatilis*	3	0.13	1	730,000
*Tryngitessubruficollis*	2	0.19	1	56,500
*Turdusmigratorius*	29	0.19	1	310,000,000
*Vermivorachrysoptera*	5	0.00	1	410,000
*Xenuscinereus*	5	0.00	1	550,000
*Zenaidamacroura*	14	0.03	1	120,000,000
*Zonotrichiaalbicollis*	27	0.13	1	140,000,000
